# Effects of Foliar Dressing with Chemical Nano-Selenum and Na_2_SeO_3_ on the Antioxidant System and Accumulation of Se and Bioactive Components in *Cyclocarya paliurus* (Sweet Tea Tree)

**DOI:** 10.3390/ijms25137433

**Published:** 2024-07-06

**Authors:** Xiaoling Chen, Qinghui Xia, Zijue Wang, Yulan Dong, Xingxing Dong, Shaopeng Zhang, Shuiyuan Cheng

**Affiliations:** 1 School of Modern Industry for Selenium Science and Engineering, Wuhan Polytechnic University, Wuhan 430023, China; chenxl0811@whpu.edu.cn (X.C.); 15571778723@163.com (Q.X.); 13039522428@163.com (Z.W.); 17603983279@163.com (Y.D.); shaopeng@whpu.edu.cn (S.Z.); 12316@whpu.edu.cn (S.C.); 2 National R&D Center for Se-Rich Agricultural Products Processing Technology, Wuhan Polytechnic University, Wuhan 430023, China; 3 School of Life Science and Technology, Wuhan Polytechnic University, Wuhan 430023, China

**Keywords:** Che-SeNPs, sodium selenite, bioactive components, antioxidant, Se species

## Abstract

Selenium (Se)-rich *Cyclocarya paliurus* is popular for its bioactive components, and exogenous Se fortification is the most effective means of enrichment. However, the effects of exogenous Se fortification on the nutritional quality of *C. paliurus* are not well known. To investigate the nutrient contents and antioxidant properties of *C. paliurus* following Se treatment, we used a foliar spray to apply Se in two forms—chemical nano-Se (Che-SeNPs) and sodium selenite (Na_2_SeO_3_). Sampling began 10 days after spraying and was conducted every 5 days until day 30. The Se, secondary metabolite, malondialdehyde contents, antioxidant enzyme activity, Se speciation, and Se-metabolism-related gene expression patterns were analyzed in the collected samples. Exogenous Se enhancement effectively increased the Se content of leaves, reaching a maximum on days 10 and 15 of sampling, while the contents of flavonoids, triterpenes, and polyphenols increased significantly during the same period. In addition, the application of Se significantly enhanced total antioxidant activity, especially the activity of the antioxidant enzyme peroxidase. Furthermore, a positive correlation between the alleviation of lipid peroxidation and Se content was observed, while methylselenocysteine formation was an effective means of alleviating Se stress. Finally, Na_2_SeO_3_ exhibited better absorption and conversion efficiency than Che-SeNPs in *C. paliurus*.

## 1. Introduction

Selenium (Se) is an essential micronutrient in humans and animals. It plays a crucial role in various biological processes [1]. In-depth research on its biological functions has demonstrated that Se can be either toxic or beneficial depending on its concentration. The World Health Organization recommends a healthy adult Se intake of 50–200 μg/day, and the Chinese Nutrition Society recommends a minimum Se intake of 60 μg/day for adults [2]. Se cannot be synthesized by the human body and can only be absorbed through Se-containing food or supplements. Plants are the main source of Se intake for humans and are important in transforming inorganic Se into its organic form through the “soil–plant–human” cycle. There is currently no evidence that Se is an essential trace element in plants, but many studies have shown that the element has a positive effect on enhancing stress tolerance, stimulating the antioxidant defense system, regulating metabolic pathways, and promoting plant growth and development [1,3,4,5].

Foliar fertilization with inorganic Se is used in the production of fruit, vegetable, and cereal crops [6]. This fertilization method displays some significant advantages over soil fertilization. For example, it allows quick supplementation of the nutrients required by crops, especially in the later stages of plant growth or when root vitality declines. Furthermore, foliar fertilization is not limited by soil conditions, enables efficient transfer of the fertilizer to the plant, and is easy to carry out, saving time and effort. More importantly, foliar fertilization does not pollute the soil. Therefore, in certain situations, foliar fertilization is more efficient and convenient than soil fertilization. Foliar spraying is used to supplement many plants. The production of moderately to highly Se-rich soybeans through foliar application of Se has been explored, as well as the main forms of Se that accumulate in soybeans at high application rates and the potential for the biological transformation of soybean crops [7]. Foliar spraying can also be used with fertilizers other than Se. For example, Eghlima et al. used foliar spraying to investigate the effect of a horsetail extract on the growth characteristics, essential oil yield, and chemical composition of basil [8].

Currently, nano-Se and sodium selenite (Na_2_SeO_3_) are popular forms of Se used in fertilizers. Due to their different absorption and transformation efficiencies in plants, these two forms of Se have their own advantages and disadvantages. Nano-Se can affect the jasmonic acid and salicylic acid biosynthesis pathways and regulate the synthesis of secondary metabolites, thereby improving the antioxidant capacity of plants, inducing crop resistance to pests and diseases while improving the quality of agricultural products [9]. In studies on rice, it has been confirmed that foliar spraying of nano-selenium can effectively improve rice quality and alleviate the accumulation of heavy metals and alleviate oxidative stress caused by heavy metal accumulation [10]. The same result also appeared in the study on wheat, which was related to the synergetic regulation of the cell wall biosynthesis pathway, uptake transporters, and antioxidative system via signaling pathways [11]. So far, there have been documented reports regarding the influence of sodium selenite and sodium selenate on the yield and selenium (Se) content of crops [12,13] and fruits [14], achieved through soil fertilization and foliar spraying methods. The results showed that the effects of selenium application on plant Se content, yield, and quality were influenced by various factors such as the method of Se application, the dose of Se applied, and the timing of Se application. In addition, the foliar application of Na_2_SeO_3_, a natural antioxidant, enables plants to absorb Se directly, thereby rapidly increasing their Se content. This not only helps to improve the nutritional value and safety of crops, but also enhances their stress resistance and yield [6].

*Cyclocarya paliurus*, also known as sweet tea tree, is a medicinal plant that only exists in China. It has gained increased attention due to its unique pharmacological properties and potential health benefits [15,16]. Polysaccharides, triterpenes, and other compounds extracted from *C. paliurus* are pharmacologically valuable for their antioxidant, anti-diabetes, and anti-hyperlipidemic properties, among others [17,18,19,20]. To date, research on Se-rich *C. paliurus* has focused mainly on the compounds that are available from its extracts [21,22,23]. However, there are few relevant reports on the methods of cultivating Se-rich *C. paliurus* and the effects of Se application on the various nutritional indicators and antioxidant capacity of these plants. In this study, we investigated the effects of foliar spraying with different forms of Se, specifically Na_2_SeO_3_ and chemical nano-Se (Che-SeNPs), on the nutritional quality of *C. paliurus*. The research focused on assessing the changes in Se content, secondary metabolite concentrations, antioxidant enzyme activities, and the expression of Se-metabolism-related genes. Additionally, the study examined Se speciation in treated leaves to gain a deeper understanding of Se utilization and metabolism in *C. paliurus*.

## 2. Results

### 2.1. The Effects of Na_2_SeO_3_ and Che-SeNPs on the Se Concentration in C. paliurus

**Figure 1:**
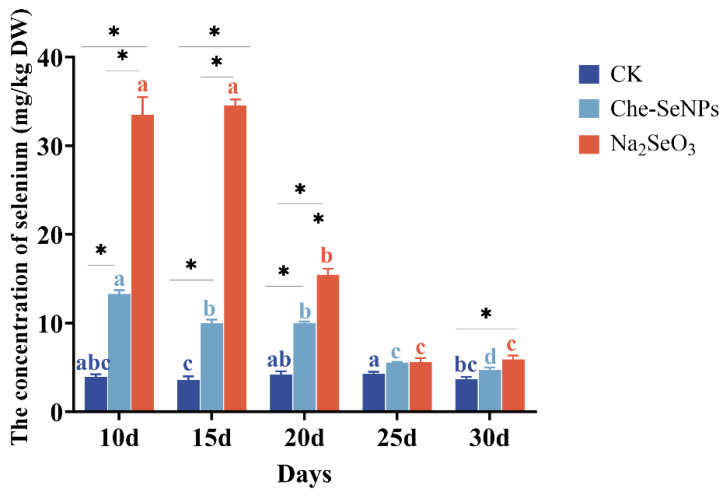
The effects of exogenous treatment with sodium selenite (Na_2_SeO_3_) and chemical nano-Se (Che-SeNPs) on the Se concentrations of *C. paliurus*. Note: using the Duncan test (*p* < 0.05), the letters of the same color indicate the degree of significant difference between the same Se source at different sampling time periods; * indicates a significant difference between different Se sources at the same sampling time period. The bars indicate the standard deviation for the three replicates. CK = control.

Compared with the control group, the Se concentrations in *C. paliurus* plants treated with Na_2_SeO_3_ or Che-SeNPs were significantly increased throughout all sampling stages (Figure 1). The highest Se concentration was obtained in the Na_2_SeO_3_ treatment group, reaching a maximum of 34.6 mg/kg at 15 days after treatment. The Se concentration in the group treated with Che-SeNPs reached a maximum of 13.3 mg/kg, one-third of the maximum seen in the Na_2_SeO_3_ treatment group, on the 10th day after treatment. Subsequently, the Se concentration in both groups showed a gradually decreasing trend.

### 2.2. The Effects of Na_2_SeO_3_ and Che-SeNPs on the Concentrations of Secondary Metabolites in C. paliurus

**Figure 2:**
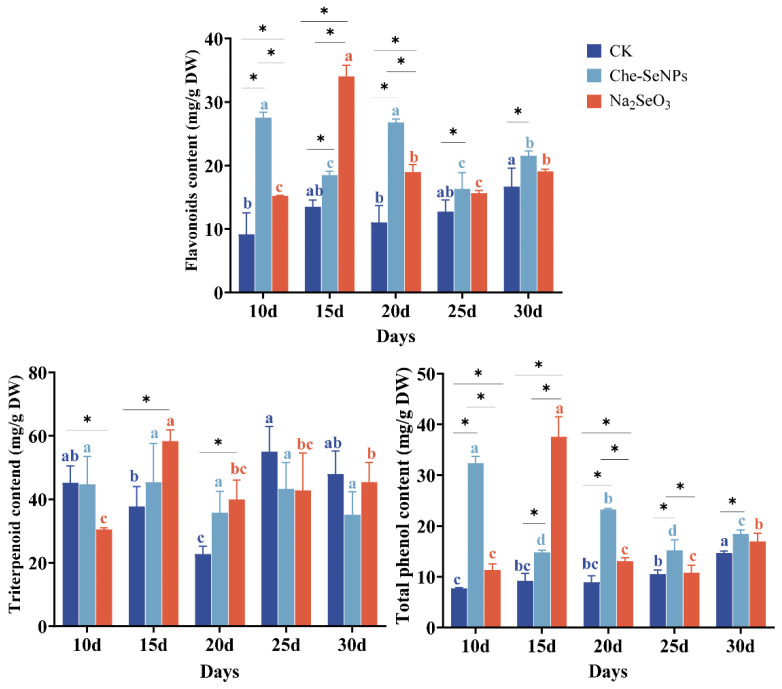
The effects of exogenous treatment with Na_2_SeO_3_ and Che-SeNPs on the concentrations of secondary metabolites in *C. paliurus*. Note: using the Duncan test (*p* < 0.05), the letters of the same color indicate the degree of significant difference between the same Se source at different sampling time periods; * indicates a significant difference between different Se sources at the same sampling time period. The bars indicate the standard deviation for the three replicates. DW = dry weight; CK = control.

Spraying different formulations of Se on *C. paliurus* leaves increased the concentrations of flavonoids, triterpenoids, and total phenol significantly. As shown in Figure 2, the flavonoid and total phenol concentrations in the Se treatment groups were significantly higher than those in the control group throughout the entire sampling period, and the triterpenoid concentration was higher under Na_2_SeO_3_ treatment than in the control group on days 15 and 20. The concentrations of flavonoids, triterpenoids, and total phenols reached their maximum values (34.03 mg/g, 58.36 mg/g, and 37.54 mg/g, respectively) at 15 days after treatment with Na_2_SeO_3_. These values were 2.5-, 1.54-, and 4-fold higher than those in the control group, respectively. At other sampling times, Che-SeNP treatment seemed to show better performance in enhancing the concentrations of secondary metabolites.

### 2.3. The Effects of Che-SeNPs and Na_2_SeO_3_ on the Antioxidant Activity of C. paliurus

**Figure 3:**
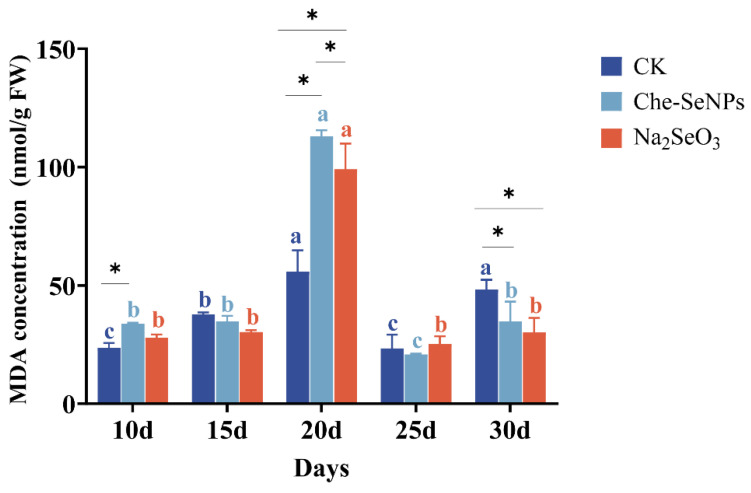
The effects of exogenous treatment with Na_2_SeO_3_ and Che-SeNPs on the malondialdehye (MDA) contents of *C. paliurus*. Note: using the Duncan test (*p* < 0.05), the letters of the same color indicate the degree of significant difference between the same Se source at different sampling time periods; * indicates a significant difference between different Se sources at the same sampling time period. The bars indicate the standard deviation for the three replicates. FW = fresh weight; CK = control.

As shown in Figure 3, the malondialdehyde (MDA) content of the three groups fluctuated throughout the sampling period, with each group showing similar change trends. The maximum values all occurred at 20 days after treatment, at which point the values in the treatment groups were twice as high as that in the control group. Over the sampling period, the average MDA content in the Che-SeNP treatment group was higher than that in the Na_2_SeO_3_ treatment group except at 25 days post-treatment, with average values of 113.0 nmol/g for the Che-SeNP group, 99.1 nmol/g for the Na_2_SeO_3_ treatment group, and 55.9 nmol/g for the control group.

### 2.4. The Effects of Che-SeNPs and Na_2_SeO_3_ on the Malondialdehyde Content of C. paliurus

**Figure 4:**
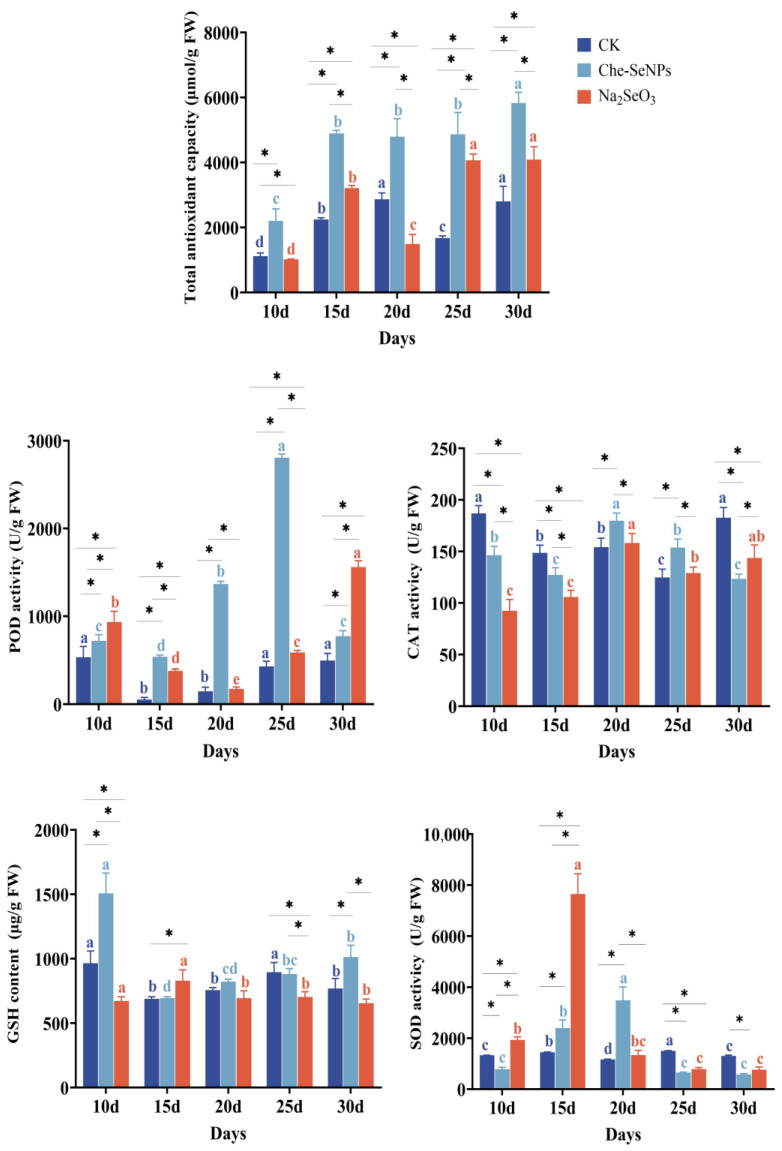
The effects of exogenous treatment with Na_2_SeO_3_ and Che-SeNPs on the antioxidant enzyme activity in leaves of *C. paliurus*. CAT = catalase; GSH = glutathione; POD = peroxidase; SOD = superoxide dismutase. Note: using the Duncan test (*p* < 0.05), the letters of the same color indicate the degree of significant difference between the same Se source at different sampling time periods; * indicates a significant difference between different Se sources at the same sampling time period. The bars indicate the standard deviation for the three replicates. FW = fresh weight; CK = control.

Different exogenous Se treatments had significant effects on the antioxidant activity of *C. paliurus*. As seen in Figure 4, the total antioxidant capacities in the treatment groups were generally greater than that in the control group. In particular, those of the Che-SeNP treatment group were 2.00-, 2.17-, 1.67-, 2.90-, and 2.08-fold higher than the control group throughout the sampling period. The same trend was found following the analysis of peroxidase (POD) activity. The glutathione (GSH) content of *C. paliurus* leaves in the Che-SeNP treatment group was elevated by 56% and 31.8% at 10 and 30 days after Se application, respectively, showing a significant difference from the control group (*p* < 0.05). At 10, 15, and 30 days after Se treatment, the catalase (CAT) activity significantly decreased compared with that of the control group. After 20 and 25 days of treatment, only the Che-SeNP treatment group showed a slight increase compared with the control group. The activity of superoxide dismutase (SOD) reached its highest value, 5.3-fold that of the control group, at 15 days after treatment with Na_2_SeO_3_ and increased to 3-fold that of the control group at 20 days after treatment with Che-SeNPs.

### 2.5. Effect of Se Speciation in C. paliurus

As shown in Table 1, foliar spraying with different Se sources had a large effect on the forms and contents of Se that accumulated in the leaves of *C. paliurus*. Methylselenocysteine (MeSeCys) was the most detectable organic form of Se in the *C. paliurus* samples. Selenocysteine (SeCys_2_) was present only in the control and Che-SeNP treatment groups, and the content in the control group was higher than that in the treatment group. Se^4+^, MeSeCys, and selenomethionine (SeMet) reached their maximum values, 2.31 μg/g, 3.41 μg/g, and 0.21 μg/g, respectively, at 10 days after Na_2_SeO_3_ treatment, exhibiting a trend of first decreasing, then increasing, and then decreasing again over time. In the Che-SeNP treatment group, the content of MeSeCys and Se^4+^ showed a trend of first increasing and then decreasing.

### 2.6. Relative Gene Expression

**Figure 5:**
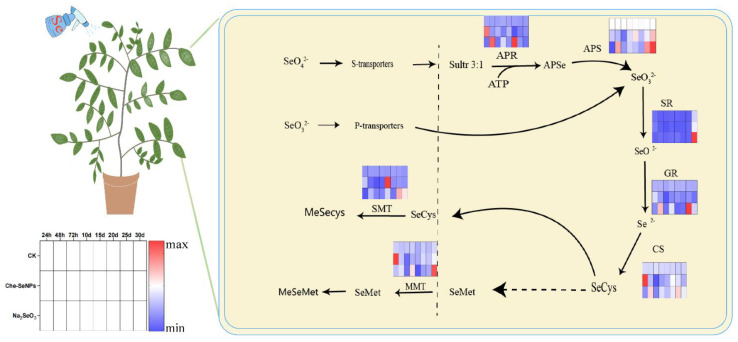
Effects of exogenous treatment with Na_2_SeO_3_ and Che−SeNPs on relative gene expression in leaves of *C. paliurus*. Note: APR: adenosine 5′-phosphoselenate reductase; APS: ATP sulfurylase; SR: sulfite reductase; GR: glutathione reductase; CS: cysteine synthase; SMT: selenocysteine methyltransferase; MMT: S-adenosyl-L-Met: Met-S-methyltransferase; CK = control.

Seven genes related to Se metabolism exhibited different expression patterns according to the Se species applied and the sampling time as shown in Figure 5. The expression of each gene in the control group remained relatively stable throughout the sampling period. In the Che-SeNP treatment group, the expression levels of APR (adenosine 5′-phosphoselenate reductase), GR (Glutathione reductase), CS (Cysteine synthase), and MMT (S-adenosyl-L-Met: Met-S-methyltransferase) were at their highest at the first sampling time point (24 h) after the application of Se; both the APS (ATP sulfurylase) and GR (glutathione reductase) genes reached their maximum values of expression at the last sampling node, specifically on day 30. Only the SMT (selenocysteine methyltransferase) gene showed maximum expression on the 20th day of sampling. Compared with the Che-SeNP treatment group, gene expression in the Na_2_SeO_3_ treatment group was more dynamic, with maximum gene expression often occurring at later sampling stages. The expression of SR was stable and low in the different groups at the various sampling time points, although there was an increase in expression at 30 days.

## 3. Discussion

### 3.1. The Effect of Se on Secondary Metabolites in C. paliurus

Both the Se and secondary metabolite contents are very important indicators of *C. paliurus* crop quality, and there is a close relationship between the contents of these indicators. Se can promote the synthesis of primary metabolites such as proteins and polysaccharides in plants, providing a material basis for the transformation of secondary metabolites. In addition, Se promotes the synthesis of key enzymes for secondary metabolite synthesis, thereby increasing the accumulation of effective substances [24]. Some studies have even found that Se can combine with secondary metabolites to form substances with unique biological activities, such as selenoflavonoid [25,26]. Se can bind to phenolic compounds and flavonoids to form Se-containing complexes. These complexes may exhibit enhanced antioxidant, anti-inflammatory, and anticancer activities compared with the individual metabolites alone. In this study, during the sampling period, foliar application of Se increased the total flavonoid and polyphenol contents in the leaves of *C. paliurus*, while the total triterpenoid content showed no significant difference from the control group during most of the experiment. It is noteworthy that in samples collected on day 15, the contents of the three secondary metabolites increased to the greatest extent compared with the control group, while the Se content in the treatment groups also reached its maximum value at this time. The results showed that the application of Se not only increased the accumulation of Se in plants, but also increased the content of secondary metabolites. In strawberries, the application of Se increased the content of flavonoid and polyphenol compounds [27], while in lettuce, the application of Se could also be attributed to the production of flavonoid glycosides and phenylpropanoic acid esters [28]. The reason for this result may be that Se is not an essential trace element for plants. When the concentration of Se reaches a certain level, it may cause a certain degree of stress in plants, and the increase in secondary product content is an effective means of resistance to this stress.

In ginkgo leaf, the application of Se can significantly increase flavonoid and lactone contents, but the mechanism of this effect is currently unclear [29]. Some studies suggest that Se may regulate the activities of flavonoid and lactone-related enzymes, thereby affecting the synthesis of flavonoids and lactones. Li et al. carried out a related study on the Se content and the nutritional quality of blueberries using foliar application of Se, and the results demonstrated that the Se content of the Se-treated group was significantly elevated by 1.5–2.3-times [30], and similar results have been obtained in tea leaves [31]. These reports, along with the results of the present study, show that spraying exogenous Se on leaves is an effective method for improving the quality of economic crops. The Se content changes seen in the present study revealed that foliar treatment with Na_2_SeO_3_ treatment is more biologically efficient than treatment with Che-SeNPs.

### 3.2. Effects of Se on the Antioxidant System of C. paliurus

Plants are constantly producing reactive oxygen species (ROS). Excessive ROS can lead to oxidative stress and cell damage, while antioxidant systems can clear excess ROS and maintain a balance of ROS levels, thereby protecting cells from oxidative stress. As an indicator of the lipid peroxidation response in plants, the level of MDAs can accurately reflect the degree of lipid peroxidation over time, thus indicating the level of the plant’s response to stress [32]. In this study, as the Se content decreased sharply 20 days after treatment, the MDA content showed a sharp increase, indicating that excessive Se had caused stress. With the extension of treatment time, both the Se content and MDA content decreased, and the MDA contents of the Se-treated groups were significantly lower than in the control group at 30 days after Se application. It indicates that a suitable content of Se has a positive effect on plants, similar to the findings reported by Li et al. [33]

This reciprocal relationship is crucial for maintaining normal physiological functions in organisms. The components of the antioxidant system are divided into two categories: enzymes and non-enzymes. Enzymes include SOD, CAT, POD, GSH-Px, etc., while non-enzymes include GSH, ascorbic acid, proline, flavonoids, alkaloids, carotenoids, etc. Se plays a very important role in the antioxidant system of plants. Se can enhance the activity of antioxidant enzymes and induce the synthesis of non-enzymatic antioxidants. In addition, it can induce the dismutation of O_2_^−^ to produce H_2_O_2_, which is then decomposed by antioxidant enzymes. The GSH content in the Che-SNP treatment group was significantly higher than that in the Na_2_SeO_3_ treatment group. This result indicated that Che-SNP treatment is more effective than Na_2_SeO_3_ with regard to GSH synthesis. Not only that, but in terms of total antioxidant activity, Che-SNP treatment also showed significant advantages compared with the Na_2_SeO_3_ and control treatments. As for other enzyme activities, there was no certain regularity, indicating that the activities of various antioxidant enzymes in plants are affected by multiple factors, further confirming that the antioxidant system is complex and comprehensive.

In summary, Se can promote the antioxidant activity of plants, and this is closely related to the species of Se applied. Similar results have been found in other studies. In a study on the effect of exogenous Se on browning in fresh-cut apples, Wang et al. [34] demonstrated that exogenous Se could significantly increase POD activity 1 month after Se application; furthermore, with the elevation of POD, antioxidant activity was stronger, and the browning degree of the fresh-cut apples was lower. Wang et al. [35] also conducted a study on the growth of maize seedlings under foliar application of Se and found that exogenous Se significantly increased plant antioxidant capacity in maize seedlings compared with the control. These findings indicate that SeNPs have the potential to augment both enzymatic and non-enzymatic components within the antioxidant defense system, thereby mitigating plant damage.

### 3.3. Molecular Mechanism of Organic Se Transformation in C. paliurus

The effect of the foliar application of different forms of Se on the organic Se content of plants is mainly reflected in the absorption, transformation, and accumulation of Se. Selenate is more easily absorbed than selenite, while elemental Se is not easily absorbed by plants. This is mainly due to the similarity between selenate, selenite, and sulfate or sulfite, which leads to competition for absorption between Se and sulfur. Therefore, although the Se content in the Se treatment groups was much higher than that in the control group, the Se content in the Che-SeNP treatment group was much lower than that in the Na_2_SeO_3_ treatment group, which fully confirmed that Na_2_SeO_3_ is better absorbed and utilized by plant leaves than nano-Se. The content of SeCys_2_ was higher in the control and Che-SeNP groups, which had lower total Se contents, indicating that SeCys_2_ is a common form of Se in the leaves of *C. paliurus*. When the Se content increased to a certain level, SeCys_2_ transformed into other forms of Se. At the start of Se application, the content of MeSeCys in *C. paliurus* rapidly increased, indicating that *C. paliurus*, like most plants, uses MeSeCys to reduce the potential toxicity of Se. The key enzyme gene SMT involved in this reaction indeed showed higher expression in the Na_2_SeO_3_ treatment group. MeSeCys can be further transformed into volatile dimethyldiselenide (DMDSe), which is an important reason why plants can tolerate high levels of Se. The APR and APS genes are key enzyme genes involved in the process of converting SeO_4_^2−^ to SeO_3_^2−^. The active expression of these two genes at certain times indicated that when Na_2_SeO_3_ is sprayed on the leaves, some SeO_3_^2−^ is oxidized to SeO_4_^2−^ and then absorbed by the leaves. SR is an enzyme gene involved in the process of converting SeO_3_^2−^ to SeO^2−^. Except at the 30-day time point, its gene expression remained stable at a relatively low level, indicating that in the case of *C. paliurus*, this process is likely to involve non-enzymatic reactions.

## 4. Materials and Methods

### 4.1. Treatment of Plant Materials

Two forms of exogenous Se were applied to the leaves of 2-year-old *C. paliurus* seedlings grown in a trial at Wuhan Polytechnic University. The Che-SeNPs were supplied by the Se Nanotechnology and Biological Effect team at Wuhan Polytechnic University. Na_2_SeO_3_ was provided by Alfa Aesar Chemical Co., Ltd. (Shanghai, China). ddH_2_O was used in the control group (CK). The selenium content in the sodium selenite and chemical nano-selenium solutions for foliar spraying is both 1 mM (previous studies have shown that under 160 mg/L Na_2_SeO_3_ (≈1 mM) treatment, *C. paliurus* can ensure the highest Se content while suffering slight damage, and all nutritional indicators perform at a good level.).

The leaves of *C. paliurus* were collected 10, 15, 20, 25, and 30 days after treatment with exogenous Se (foliar spraying method). The experiment started in September 2022. Five individuals were considered as one replicate, and each group had three biological replicates. The collected samples were divided into two sets. One set was dried at 65 °C to a constant weight and then crushed into powder for future use. The other set was rapidly frozen in liquid nitrogen and stored in a −80 °C freezer.

### 4.2. Determination of Se and Secondary Metabolites Content in Leaves of C. paliurus

The total Se content was measured by hydride generation atomic fluorescence spectrometry (HG-AFS). A 0.2 g sample of the dried leaf powder was weighed accurately and placed into a 35 mL digestion tube; 7 mL 65% HNO_3_ was added, and the tube was placed in an acid digestion vessel for pre-digestion at 180 °C until there was little yellow smoke. The tube was then placed in a microwave digestion system (default procedure). After the acid removal step, about 1 mL of residue was left in the tube. The residue was diluted to 10 mL with 10% HCl to be measured by HG-AFS (AFS8510, Haiguang Instrument, Beijing, China).

The contents of Se species were determined by inductively coupled plasma mass spectrometry (ICP-MS). A total of 5 mg of protease E and 5 mg of protease K were mixed with 3 mL water, respectively. A 0.1 g sample of dry leaf powder was mixed with 1.5 mL protease K solution and placed in a 10 mL centrifuge tube. The tube was then placed in a water bath for ultrasonic enzymatic hydrolysis. After the reaction, the mixture was centrifuged (10,000 rpm, 20 min), and the supernatant was filtered through a 0.22 μm organic filter membrane before being analyzed on an ICP-MS system (8900, Agilent, Santa Clara, CA, USA).

The contents of three secondary metabolites, including flavonoids, triterpenoids, and polyphenols, were determined according to methods described by Zhang et al. [36], Xia et al. [37], and Qin et al. [38]

### 4.3. Determination of Antioxidant System

The contents of MDA and reduced GSH were determined using kits (KTB1050-48T) from Abbkine Scientific Co., Ltd. (Wuhan, China) and (AKPR008M) Box Biotechnology Co., Ltd. (Beijing, China). The activities of POD (EC 1.11.1.7), SOD (EC 1.15.1.1) and CAT (EC 1.11.1.6) were determined using kits [KTB1640-48T (GSH-Px), KTB1030-48T (SOD), KTB1040-48T (CAT), KTB1150-48T (POD), KTB1500-48T (TAC)] from Abbkine Scientific Co., Ltd. (Wuhan, China).

### 4.4. Screening and Expression Pattern Analysis of Se-Metabolism-Related Genes

Based on the *C. paliurus* genomic and transcriptome data, seven key enzyme genes involved in Se metabolism in *C. paliurus* were screened. Specific primers were designed by Primer Premier 5.0 (Table 2). *18s ribosomal RNA* was used as the endogenous reference gene. The experimental procedures, reagents, and data analysis methods were as described by Xia et al. (2023) [37].

## 5. Conclusions

This study delved into the effects of foliar spraying of different forms of Se on the nutritional quality of *C. paliurus* leaves. The experimental results showed that different forms of Se had significant effects on the Se content, antioxidant enzyme activity, and accumulation of bioactive components in *C. paliurus* leaves, as well as the forms of Se found in the leaves. Firstly, the application of Se significantly increased the Se content in leaves along with the content of organic Se, and spraying with Na_2_SeO_3_ enriched the Se content more efficiently than spraying with Che-SeNPs, due to the higher absorption, transportation, and utilization efficiency of Na_2_SeO_3_ in the leaves of *C. paliurus*. Secondly, foliar spraying of Se significantly enhanced the content of secondary metabolites and antioxidant enzyme activity in the leaves of *C. paliurus*, indicating that Se can enhance the resistance of *C. paliurus* to stress and improve its antioxidant capacity.

These results indicated that foliar spraying with Se can not only increase the Se content in the leaves of *C. paliurus*, but also improve its overall nutritional quality. This study provides new ideas and methods for the regulation of Se in *C. paliurus*, which is of great significance for improving the nutritional value and health functions of *C. paliurus* products. Furthermore, the results provide a valuable reference for the study of Se nutrition in other plants.

## Figures and Tables

**Table 1:** Effects of foliar spraying with different Se sources on Se morphology and content in leaves of *Cyclocarya paliurus*.

Treatment	Sampling Time (d)	Content of Different Se Forms (μg/g DW)			
		SeCys_2_	MeSeCys	Se^4+^	SeMet
CK	10	0.01329 ± 0.00060a	0.01689 ± 0.00059b	0.01464 ± 0.00390b	—
Che-SeNPs		0.00261 ± 0.00028b	0.11256 ± 0.00581b	0.07568 ± 0.00640b	0.01582 ± 0.06102c
Na_2_SeO_3_		—	3.41292 ± 0.85489a	2.31044 ± 0.57598a	0.02058 ± 0.03565b
CK	15	0.01015 ± 0.01544a	0.04717 ± 0.00230d	0.03159 ± 0.00116d	—
Che-SeNPs		—	0.14418 ± 0.00860a	0.09492 ± 0.00627a	—
Na_2_SeO_3_		—	0.06318 ± 0.00383b	0.04130 ± 0.00148b	—
CK	20	—	0.01422 ± 0.02915d	0.01311 ± 0.01399d	—
Che-SeNPs		—	0.14723 ± 0.00411b	0.09673 ± 0.00517b	—
Na_2_SeO_3_		—	1.29495 ± 0.05173a	0.86270 ± 0.03334a	0.00377 ± 0.00653a
CK	25	—	0.03250 ± 0.03041b	0.03093 ± 0.00198b	—
Che-SeNPs		—	0.00968 ± 0.01113c	0.00941 ± 0.00041c	0.00306 ± 0.00521a
Na_2_SeO_3_		—	0.07881 ± 0.00618a	0.05159 ± 0.00500a	0.00218 ± 0.00475b
CK	30	0.00918 ± 0.00363a	—	0.0012 ± 0.00536a	0.00501 ± 0.00295b
Che-SeNPs		0.00024 ± 0.00112b	—	—	0.00333 ± 0.01032c
Na_2_SeO_3_		—	—	—	0.00984 ± 0.01277a

Note: lowercase letters indicate significant difference according to Duncan’s test between Se sources at the same sampling time. DW = dry weight; CK = control.

**Table 2:** Primer sequences for qRT-PCR reaction.

Primer Name	Primer Sequence (5′ to 3′)
*18s*	F: AGTATGGTCGCAAGGCTGAAA
	R: CAGACAAATCGCTCCACCAA
*APR*	F: GACCGCACTCATTCTCTATC
	R: TCCTCCACCTCTACCTTCT
*APS*	F: GTCGTCGGCTTCTTGAGATG
	R: TCGTAGTAGTCAACCAGCACTT
*CS*	F: CTCCAGGAAGGCTTGTTG
	R: GCACGCTACGGATGATAG
*GR*	F: GTGGAACTTGACGAGACAG
	R: CAACTGCCTGCTCTTCAC
*MMT*	F: AAGCCTTCTCAGACTTGGTAG
	R: ACTGATAGTGGCACAGAATT
*SMT*	F: CAGAGTGTGCCTCCATTG
	R: GCTTCCGTATAGATGTAATCAG
*SR*	F: GGTCTCAGGTCCTCCAACTC
	R: CATCCGTCAACATCTCCTCATT

## Data Availability

The data presented in this study are available upon request from the corresponding author.
